# A new central venous access in Emergency Department: ultrasound-guided infraclavicular axillary vein

**DOI:** 10.1186/2036-7902-7-S1-A7

**Published:** 2015-03-09

**Authors:** M Algaba-Montes, A Oviedo-García

**Affiliations:** Emergency Department, Valme Hospital, Seville, Spain

**Keywords:** Parenteral Drug, Ultrasound Guidance, Emergency Physician, Axillary Artery, Central Venous Access

## Background

The central veins that are usually cannulated are the jugular, subclavian and femoral. As we know ultrasound guidance can reduce complication rates and increase the success of cannulation.

## Objective

We present a case of ultrasound-guided axillary vein placement catheter by Emergency Phisicians. This approach is not widely used among emergency physicians. We set an objective to spread this technique among emergency physicians because of its safety features for the patient.

## Patients and methods

A patient addicted to parenteral drugs, was admitted to the ER in clinical condition of septic shock.

## Results

52 year old woman addicted to parenteral drugs, stage C3 HIV with 2 weeks duration fever. On arrival had malaise, hypotensive, febrile, tachycardic... it was not possible to catheterize a peripheral vein we performed a central line cannulation: ultrasound-guided infraclavicular axillary vein. Less arterio-venous overlap and a greater distance between artery and vein and from vein to rib cage should provide an increased margin of safety for central venous cannulation. We will describe step by step, accompanied by images, the steps necessary to achieve infraclavicular axillary vein cannulation.

## Conclusion

The ultrasound-guided axillary approach offers a number of potential advantages over others central line cannulation. The anatomy favours ultrasound guidance and less complications. Manual compression of the axillary artery or surgical access is possible if arterial damage is caused. The puncture site is further away from potential sources of infection in patients with tracheostomy, central chest wall burns or sternotomy wounds. Once mastered, this is a safe, useful, and reliable technique for central venous access, so the axillary vein is an alternative for central venous cannulation, as can be seen in the case presented, an effective alternative to US-guided IJV and SCV cannulation.

## Informed consent

The study was conducted in accordance with the ethical standards dictated by applicable law. Informed consent was obtained from each owner to enrolment in the study and to the inclusion in this article of information that could potentially lead to their identification.
